# Microbial Community Changes in Silkworms Suspected of Septicemia and Identification of *Serratia* sp.

**DOI:** 10.3390/ijms25073957

**Published:** 2024-04-02

**Authors:** Jong Woo Park, Seul Ki Park, Chan Young Jeong, Hyeok Gyu Kwon, Ji Hae Lee, Sang Kuk Kang, Seong-Wan Kim, Seong-Ryul Kim

**Affiliations:** Department of Agricultural Biology, National Institute of Agricultural Sciences, Wanju-gun, Jeonju 55365, Jeollabuk-do, Republic of Korea; seulki4893@korea.kr (S.K.P.); duckbb1@korea.kr (C.Y.J.); hukgoo@korea.kr (H.G.K.); jihae@korea.kr (J.H.L.); wkdudghl@korea.kr (S.K.K.); tarupa@korea.kr (S.-W.K.); ksr319@korea.kr (S.-R.K.)

**Keywords:** microbial community, septicemia, *Serratia*, silkworm

## Abstract

Diseases that occur in silkworms include soft rot, hardening disease, digestive diseases, and sepsis. However, research on the causes of bacterial diseases occurring in silkworms and the resulting changes in the microbial community is lacking. Therefore, we examined the morphological characteristics of sepsis and changes in the microbial community between silkworms that exhibit a unique odor and healthy silkworms; thus, we established a relationship between disease-causing microorganisms and sepsis. After producing a 16S rRNA amplicon library for samples showing sepsis, we obtained information on the microbial community present in silkworms using next-generation sequencing. Compared to that in healthy silkworms, in silkworms with sepsis, the abundance of the *Firmicutes* phylum was significantly reduced, while that of *Proteobacteria* was increased. *Serratia* sp. was dominant in silkworms with sepsis. After bacterial isolation, identification, and reinfection through the oral cavity, we confirmed this organism as the disease-causing agent; its mortality rate was 1.8 times higher than that caused by *Serratia marcescens*. In summary, we identified a new causative bacterium of silkworm sepsis through microbial community analysis and confirmed that the microbial community balance was disrupted by the aberrant proliferation of certain bacteria.

## 1. Introduction

Insects have symbiotic relationships with various microorganisms [1], and the interactions between insects and microorganisms can range from complementary to pathogenic [2]. In particular, the various microorganisms present in the intestines of insects affect their nutrient supply [3], digestion [4,5], and defense against external stimuli [1]. However, in special circumstances, these microorganisms present in the intestines act as pathogens that produce virulence factors depending on environmental changes [6]. For example, in *Manduca sexta* (Linnaeus), when *Enterococcus* present in the midgut spreads to the hemolymph, pathogenicity occurs [4]. In addition, when *Enterobacter cloacae*, which is commonly found in the intestines of insects, is administered orally to *Spodoptera litura* (Fab.), it causes pathogenicity through alterations in the intestinal microbial community [7]. Oral administration of *Flavobacterium* spp., *Klebsiella* spp., and *Serratia marcescens* has been confirmed to be pathogenic to insects [8]. Thus, while the gut microbiota in insects has a positive impact on nutrient supply, digestion, and defense against external stimuli, changes in the gut microbial community can affect the growth and development of insects, which threatens to cause economic losses and endanger public health and, therefore, implies a need for disease management related to industrially produced insects. Therefore, understanding the microbiome and its impact is important in microbe-induced insect diseases for disease prevention and control.

Silkworms (*Bombyx mori*), which belong to the family Bombycidae and order Lepidoptera of class Insecta, have mainly been previously used in the textile industry. However, in Korea, as the natural fiber industry has declined owing to the development of artificial fibers, research on the use of silkworms as functional food materials has become active [9,10]. For example, just before forming a cocoon, silkworms produce compounds with various functions, such as preventing Alzheimer’s disease, lowering blood sugar levels, and lowering cholesterol; hence, they are attracting attention as raw materials for functional health foods [10,11]. When using silkworms as functional foods, microbial contamination should be managed to improve productivity and ensure hygiene and safety. Although many studies have been conducted on the fungi [12,13] and viruses [14] that cause diseases in silkworms, there is a lack of research on bacterial diseases to isolate and identify new disease-causing bacteria other than *Bacillus thuringiensis* and *S. marcescens* [8,15].

In the growing insect industry, entomopathogenic bacteria can quickly and easily infect and multiply, resulting in the reduced production of industrial insects and subsequent economic losses [16]. Sepsis, a common bacterial disease in insects, is associated with entomopathogenic bacteria that attach to specific tissue surfaces and multiply, producing various immunosuppressive factors, toxin proteins, and specialized metabolites that cause rapid death of the host [17]. Therefore, entomopathogenic bacteria are often used as biological control agents to suppress pests in plant cultivation. Representative entomopathogenic bacteria include *Bacillaceae*, *Pseudomonadaceae*, *Enterobacteriaceae*, *Streptococcaceae*, *Micrococcaceae*, and *Paeni-bacillaceae*; additionally, *S. marcescens* and *S. entomophila* are attracting attention for use as biological control agents owing to their strong pathogenicity [16,17]. *S. marcescens* and *S. entomophila* infect lepidopteran insects, impairing their feeding and growth, and rapidly proliferate and secrete large amounts of proteolytic enzymes such as β-hemolysins, elastases, and chitinases. The secreted proteases also aid the pathogenic bacteria in their transition from the gut to the hemolymph, where they destroy the humoral immune system, leading to death within several hours after infection. While it has the advantage as a biological control agent due to its strong and rapid infectious ability, it can also cause serious economic losses against industrial insects [16]. As such, the study of bacterial diseases in insects can have several purposes, underlining the importance of the isolation and identification of new microorganisms that exhibit strong pathogenicity in insects.

Therefore, in this study, we analyzed the microbiome of septicemic silkworms to select dominant bacteria; furthermore, we isolated and identified bacteria expected to be the cause of septicemia using Koch’s postulates test. Our study is significant as we isolated a new potent entomopathogenic bacterium that could be used as a biopesticide and as a basis for the development of gene amplification markers for early diagnosis of sepsis, which causes considerable economic losses in the silkworm industry.

## 2. Results

### 2.1. Morphological Diagnosis of Silkworm Sepsis

**Figure 1:**
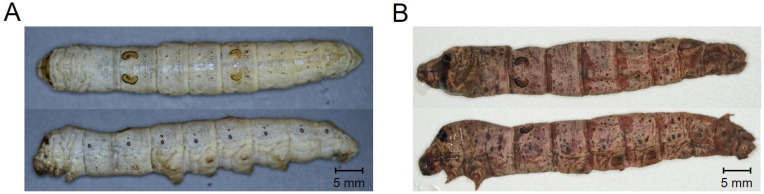
Morphological comparison between healthy and septic silkworms. (**A**) Healthy silkworms have a white and elastic body. (**B**) Septic silkworms had small brown spots on their red bodies and lost elasticity.

Figure 1 shows healthy silkworms and those diagnosed as having silkworm septicemia. Healthy silkworms were bright white, had a cylindrical body, and were elastic (Figure 1A). However, silkworms classified as septic were red in color with small brown spots scattered throughout and a flat body in an upright position (Figure 1B). In addition, healthy silkworms had no odor, and those that turned red showed morphological characteristics that were typical of sepsis caused by *S. marcescens*, a well-known causative agent of sepsis; however, they had a fouler odor than that associated with sepsis caused by *S. marcescens*. Thus, although the causative bacteria could not be identified, it was confirmed that the silkworms could be divided into two groups, a normal group and a sepsis group, based on morphological characteristics.

### 2.2. Microbial Diversity in Silkworms with Sepsis

**Figure 2:**
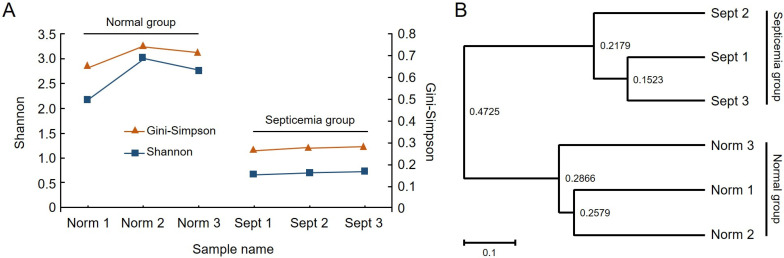
Community diversity analysis of silkworm samples diagnosed with sepsis. (**A**) Community richness and diversity changes in infected silkworm. (**B**) Unweighted pair group method with arithmetic mean tree using average linkage.

After preprocessing the raw reads obtained by next-generation sequencing, 77,753 reads were obtained on average (Tables S1 and S2), and a microbial community analysis of healthy and septic silkworms was performed using the amplicon sequence variant (ASV) method (Table S3). Good’s coverage exceeded 99%, indicating that sufficient reads had been obtained to analyze the entire bacterial community. Analysis of the species diversity (alpha-diversity) within the sample (Figure 2A) revealed that both Shannon and Gini–Simpson diversity indices decreased by >10 times in the septicemia group than in the healthy group (Table S3). An analysis of the similarity (beta-diversity) of the microbial community between samples (Figure 2B) confirmed that the communities within each group were highly related and similar; however, the correlation greatly reduced between the healthy and septicemia groups. We confirmed that they contained different microbial communities.

**Figure 3:**
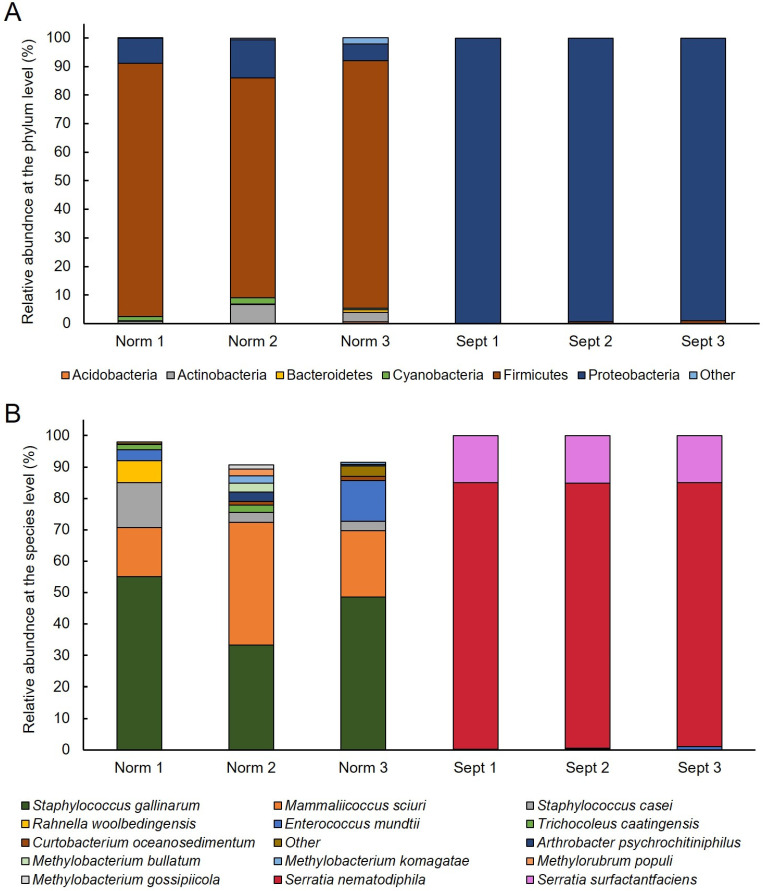
Community richness and diversity changes in infected silkworms. (**A**) Relative abundances evaluated at the phylum level and (**B**) abundances of the selected bacterial taxa (top 10 for each sample) present at the species level in two groups of silkworms. Bacterial taxa are clustered and identified by ASV analysis.

The microbial community composition of the samples within each group was analyzed by relative abundance at the phylum level, as shown in Figure 3A. As a result, 14 phyla, including an unclassified group, were identified. In the healthy group, *Firmicutes* accounted for more than 76%, whereas in the septicemia group, *Proteobacteria* accounted for more than 98% of the total bacterial population. These results indicate that the causative bacteria of septicemia are *Proteobacteria*. To identify specific septicemia-causing bacteria, 206 species were isolated in the two groups, including unidentified species. After sorting and analyzing only the top 10 species in the healthy and septicemia groups (Figure 3B), *Staphylococcus gallinarum* was found to be dominant, with an average of 45.66% in the healthy group, followed by *Mammaliicoccus sciuri*, accounting for approximately 25.3%. However, in the septicemia group, *Serratia nematodiphila* accounted for the majority (84.45%), followed by *Serratia surfactantfaciens* (15%).

### 2.3. Isolation and Identification of Bacteria from Silkworms with Sepsis

**Figure 4:**
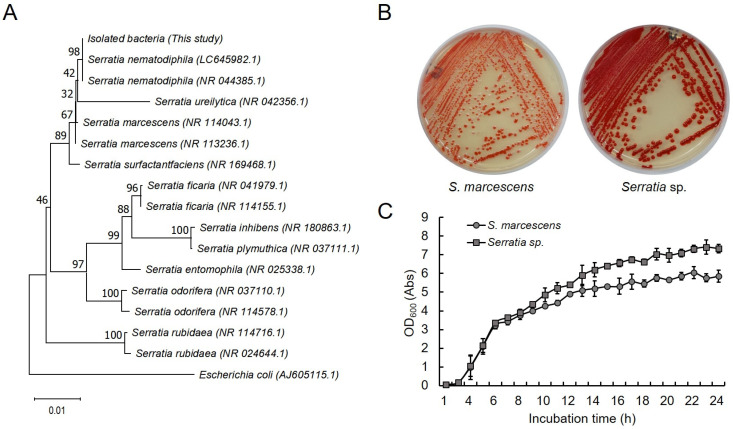
Isolation and identification of causative microorganisms from silkworms with sepsis. (**A**) Phylogenetic trees based on 16S rRNA gene sequence (the size of about 1100 bp). Sequence alignment and phylogenetic inferences were obtained using the maximum NJ within MEGA 11 software. Numbers at the nodes are percentages of bootstrap values obtained by repeating the analysis 1000 times to generate a majority consensus tree. (**B**) Comparison of the morphology of two *Serratia* sp. Both bacterial strains were cultured in the same Luria Broth (LB) medium for 18 h. (**C**) Comparison of growth patterns of two *Serratia* sp. The cells were cultured using LB medium, and the OD_600_ value was measured. Values represent mean ± SD of triplicate experiments.

To confirm whether the bacteria presumed to be the causative agent in septic silkworms were *S. nematodiphila*, we isolated and cultured the bacteria. We then aligned the sequences of the internal regions of the 27F and 1492R primers of the 16S rRNA gene and constructed a phylogenetic tree using the neighbor-joining (NJ) method (Figure 4A). We confirmed that the 16s rRNA gene sequences of the isolated bacteria were closely related to *S. nematodiphila* (LC645982.1 and NR_044385.1) and formed the same clade (98% bootstrap values). When 1148 bp of the 16s rRNA region base sequence of the isolated bacteria was aligned with the base sequences of *S. nematodiphila* (LC645982.1) and *S. marcescens* (NR_114043.1) using Clustal Omega, the isolated bacteria showed 100% sequence similarity with LC645982.1 and 99.83% similarity with NR_114043.1 (Figure S1). These results indicate that the isolated bacteria are closely related *to S. nematodiphila*. In addition, with regard to the colony formation and growth pattern (Figure 4B,C), the isolated strain formed redder and larger colonies compared to those formed by *S. marcescens*, which is generally known to cause silkworm septicemia. Thus, these are strains with different morphological characteristics. Additionally, the bacteria isolated in this study showed a rapid growth rate with a maximum absorbance approximately 1.25 times higher after 24 h of incubation.

### 2.4. Pathogenicity Verification of Isolated Bacteria

**Figure 5:**
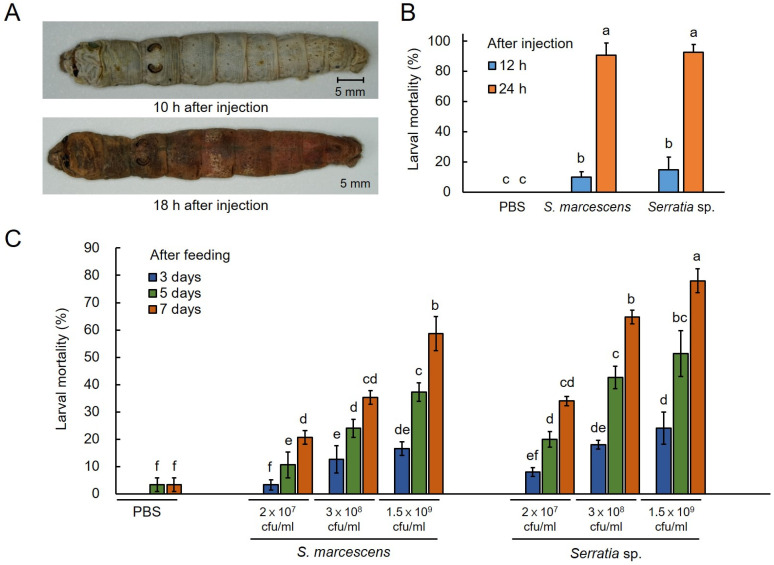
Pathogenicity verification of isolated *Serratia* sp. (**A**) Sepsis induced by isolated *Serratia* sp. injection. After subcutaneous injection of 5 × 10^5^ cfu of isolated *Serratia* sp. into 5-day instar silkworms, morphological changes over time were monitored. (**B**) Mortality following subcutaneous injection of two *Serratia* sp. When the two strains were injected at the same concentration, the mortality rate was examined 12 and 24 h later. (**C**) Mortality rate according to feeding of two *Serratia* sp. Two bacterial species were diluted to 2 × 10^7^, 3 × 10^8^, and 1.5 × 10^9^ cfu/mL and were fed to the worms once-off via mulberry leaves, and mortality was examined for 7 days. The columns and bars represent mean ± SD of triplicate experiments. Mean values with same letters above a bar are not significantly different at *p* < 0.05 by Duncan’s multiple range test.

To determine whether the isolated bacteria had insect pathogenicity similar to that of *S. marcescens*, inoculations were performed via direct injection into the body cavity or mixing with feeding, and disease induction due to infection was verified. When 5 × 10^5^ cfu of isolated *Serratia* sp. was inoculated directly into the body cavity of the silkworm, the test group developed symptoms of septicemia, including failure to feed and loss of skin elasticity, approximately 10 h after injection; at 18 h after inoculation, the epidermis turned red and the silkworms died. The morphological characteristics of the infected worms (Figure 5A) were similar to those shown in Figure 1B. The mortality rate after subcutaneous injection was not different from that of control worms infected with *S. marcescens*, and the mortality rate for both strains was >90% within 24 h (Figure 5B).

To induce a situation similar to that of a natural infection through contaminated leaves, the pathogenicity of oral infection was compared after feeding the silkworms with food that was mixed with microorganisms, as shown in Figure 5C. We diluted *S. marcescens* and isolated *Serratia* sp. to three concentrations, infected the worms by feeding them these diluted bacterial cultures via food, and monitored the mortality rate. Both strains resulted in silkworm mortality from the third day after inoculation. From the fifth day, worms infected with the isolated *Serratia* sp. had a higher mortality rate than that of worms infected with *S. marcescens*. Finally, on the seventh day, the mortality rate of worms fed *S. marcescens* was 35.33% at the intermediate concentration of 3 × 10^8^ cfu/mL, whereas that of worms fed with isolated *Serratia* sp. at the same concentration was 64%, indicating a 1.8 times higher mortality rate with isolated *Serratia* sp. Sepsis caused by bacterial inoculation through feeding showed the same symptoms as those observed after injection inoculation. Bacteria were re-isolated from the infected silkworms using the same method as that used for naturally infected pathogens and identified through sequence analysis (Figure S2). The isolates were confirmed to be isolated *Serratia* sp.

## 3. Discussion

### 3.1. Characteristics of Silkworms Showing Sepsis Symptoms

For the diagnosis and prevention of insect diseases, observation of morphological characteristics is used as the simplest and fastest method for evaluation; therefore, accurate assessment and classification of characteristics are important. Sepsis in silkworms causes death when pathogenic bacteria invade and proliferate in the silkworm’s body [18]. Representative sepsis-causing strains include *S. marcescens*, *Bacillus proteus*, *Pseudomonas* sp., *Streptococcus* sp., and *Bacillus* sp. The first symptom noted after infection is that the back of the head of the silkworm turns black, and over time, the color of the epidermis changes after death [18]. *S. marcescens* causes black-brown spots to appear all over the body immediately after death, while the body turns red over time, as was also noted in this study [19]. According to these characteristics, the silkworms in the present study would be considered to have developed sepsis caused by a typical *S. marcescens* bacterial infection. However, the septicemic silkworms collected in this study had a distinctive odor that was different from those infected with *S. marcescens*, suggesting that they may not be septicemic. Accordingly, in this study, a more precise and reliable molecular diagnostic method, i.e., microbial community analysis, was used, which can be used to analyze the cause of the disease more accurately than morphological diagnosis or culture [20].

### 3.2. Role of Microbial Community in Inducing Sepsis in Silkworms

Recently, interest in microbial communities and symbiosis between microorganisms and crops has increased. Many studies have been conducted to identify useful bacteria using microbial community analysis. Microbial community analysis is a powerful tool in disease research [20]. In a previous similar study of the intestinal microbial community of insects, numerous microorganisms were found to live in the intestines of healthy insects, with the balance disrupted under specific environmental conditions, resulting in disease onset [21,22]. According to Koch’s postulates, microbial community analysis is used in disease diagnosis because the initial condition of a disease-causing pathogen is “dominant” [23]. Ultimately, in our microbial community analysis, shown in Figure 3, silkworms grown under the same conditions showed marked differences in the microbial communities according to the presence or absence of sepsis because of differences in the immune, nutritional, and physiological factors of each individual and *S. nematodiphila* introduced from the external environment. This is believed to be because the introduction of these bacteria disrupted this balance [21]. In fact, in the microbial community analysis of healthy silkworms, *S. nematodiphila* was not present in the intestines. In addition, according to the results of the pathogenicity analysis (Figure 5), inoculation by subcutaneous injection affected physiological metabolism through direct proliferation in the hemolymph of the silkworm without affecting the intestinal microbial community and resulted in a mortality close to 100% in a short period of time. Oral infection required a longer time for the characteristics of the disease to develop, and differences were noted between individuals; therefore, the fatality rate did not reach 100%. Thus, our results further confirmed that both the microbial community existing in the intestines and the genetic and physiological characteristics of each individual play a role in disease occurrence. However, to improve our understanding of disease occurrence in silkworms, additional research on the possibility of mutual inhibition by intestinal microorganisms is required [24].

### 3.3. Identification of Causative Bacteria of Sepsis through Verification of Koch’s Postulates

According to Koch’s postulates, four conditions are necessary to establish a relationship between disease and microorganisms [23]. According to the first postulate, “microorganisms should be detected in large quantities in organisms suffering from certain diseases” [23]. As shown in Figure 3, *S. nematodiphila* was detected in large quantities in septic silkworms in our microbial community analysis; therefore, the causative agent of silkworms classified as septic was assumed to be *S. nematodiphila*. To verify whether the second hypothesis, which states that “pure isolation and single culture from disease-infected organisms should be possible” [23], was satisfied, bacteria were isolated and identified from the septic silkworms (Figure 4). The isolated and identified bacteria were classified as *S. nematodiphila* and *S. marcescens*; however, these organisms were so closely related that they could not be separated from each other based on sequence similarity alone. Studies on the identification of *Serratia* sp. have also revealed that *S. nematodiphila* and *S. marcescens* are genetically highly similar [25].

Finally, a comparison based on the culture characteristics confirmed that the bacteria isolated from the septic silkworms had characteristics different from those of *S. marcescens*. Further, a phylogenetic relationship analysis showed that the isolated bacteria had highest similarity with *S. nematodiphila*, suggesting that it could be a species of *S. nematodiphila* [26,27]. To verify Koch’s postulates 3 and 4, silkworms were artificially infected with the isolated and cultured bacteria (Figure 5). This resulted in a higher pathogenicity than that of *S. marcescens*, which was used as a control. Additionally, the organisms could be re-isolated from infected silkworms, satisfying Koch’s postulates [1]. Thus, the causative bacterium for the sepsis in silkworms in this study was determined to be *Serratia* sp., which is different from the generally implicated *S. marcescens*.

In summary, this study is significant for the discovery and identification of a new microorganism that causes sepsis. We believe that this isolate can be used in the development of markers and disinfectants to diagnose and prevent sepsis in the future. However, the species classification of *Serratia* sp. in this study was based only on partial sequencing of the 16S rRNA gene sequence, and it is important to obtain additional information on the isolated bacteria through further whole-genome sequence analysis [27].

## 4. Materials and Methods

### 4.1. Silkworm Sample Preparation

The silkworms used in this experiment were BaekokJam (Jam 123X124). BaekokJam is a silkworm strain developed and bred in 1983 by the Department of National Institute of Agricultural Science of the Rural Development Administration (Wanju-gun, Republic of Korea), which conducted this study, and is an F1 hybrid of Japanese and Chinese silkworms. Worms were fed fresh mulberry leaves and were reared at a temperature of 24–27 °C and humidity of 70–90% under a 16 h/8 h light/dark cycle. Among the reared silkworms, healthy silkworms as well as silkworms whose epidermis turned red and became soft at 6 days of the fifth instar and that had a stronger odor than that of worms with general septicemia were selected. After washing the worms with 70% ethanol, each individual was placed in an ultra-low temperature freezer at −80 °C.

### 4.2. DNA Extraction and 16S rRNA Amplicon Sequencing

Five healthy and five red silkworms were pooled separately and then pulverized for genomic DNA extraction using the MagMax Microbiome Ultra Nucleic Acid Isolation Kit (Applied Biosystems, Foster City, CA, USA) according to the manufacturer’s instructions. For the microbial community analysis, a library was created by amplifying the V3-V4 region of the 16S ribosomal RNA gene using the Nextera XT Index V2 Kit (Illumina, San Diego, CA, USA) and was analyzed using MiSeq (Illumina, San Diego, CA, USA). Sequence analysis was performed by Macrogen Co. Ltd. (Daejeon, Republic of Korea).

### 4.3. Microbial Community Analysis

The FASTQ file produced for the microbial community based on the ASV was trimmed using Cutadapt Quality (https://cutadapt.readthedocs.io/en/stable/guide.html, accessed on 29 January 2022). Filtering, denoising, merging, and chimera removal were performed using the DADA2 pipeline (https://benjjneb.github.io/dada2/tutorial.html, accessed on 29 January 2022), and 100% matching sequences were obtained. Altogether, they formed ASV. Sequence information was obtained from the NCBI 16S ribosomal RNA database (NCBI_16S_20211127 (BLAST)), and a community structure analysis was performed using the Quantitative Insights into Microbial Ecology (QIIME, version 1.9) program [26,27]. To analyze the diversity of clusters within a sample, alpha-diversity was analyzed using Chao1, Shannon, and Gini–Simpson indices, and beta-diversity was used to analyze the diversity of clusters between samples using the unweighted pair group method based on arithmetic means. Phylogenetic relationships were visualized using a phylogenetic tree and principal coordinate analysis.

### 4.4. Isolation and Identification of Microorganisms

First, 5 mL of phosphate-buffered saline (PBS) was added to 50 mg of pulverized silkworm samples for microbial community analysis, and the samples were homogenized. The homogenized suspension was then serially diluted up to 10^10^-fold, and 100 μL of each diluted sample was plated on LB, nutrient agar (NA), and tryptic soy blood agar and incubated at 27 °C for 96 h. Colonies from each plate that showed morphological differences were selected and subcultured five times to isolate a single strain. The isolated bacteria were cultured in liquid medium, after which genomic DNA was extracted using a G-spin genomic DNA extraction kit (iNtRON Biotechnology, Seongnam-si, Republic of Korea). The 16S rRNA gene was amplified by PCR using universal primers 27F (5′-AGAGTTTGATCMTGGCTCAG-3′) and 1492R (5′-GGTTACCTTGTTACGACTT-3′) [28,29]. The PCR products were sequenced at Macrogen Co., Ltd. (Daejeon, Republic of Korea), and sequence alignment was performed using the GenBank database of the NCBI (http://www.ncbi.nlm.nih.gov/, accessed on 10 January 2022). Sixteen 16S rRNA gene sequences of the genus *Serratia* were selected for the phylogenetic analysis. The 16S rRNA gene of *Escherichia coli* strain BL21 was used as an outgroup. Subsequently, the distances and clusters were analyzed using ClustalW and MEGA 11 [30], and a phylogenetic tree was created using the NJ method. The evolutionary history of the NJ method is based on the Tamura–Nei model [31]. Bootstrap values were calculated based on 1000 replicas. The identified strain was stored in 20% (*w*/*v*) glycerol at −80 °C.

### 4.5. Pathogenicity Verification of Isolated Bacteria

To verify the pathogenicity of *Serratia* sp., which was isolated and identified from silkworms showing sepsis, *S. marcescens* KACC11961 was purchased from the Korean Agricultural Culture Collection (Wanju, Republic of Korea) to be used as control. Bacteria isolated after being infected with silkworms were again tested in silkworms. A single colony of *Serratia* sp. isolated in this study was inoculated into NA medium and cultured at 27 °C for 24 h. The cultured bacteria were recovered by centrifugation at 4000× *g* at 4 °C, resuspended using PBS, and diluted to an OD600 of 1.5 (approximately 3 × 10^9^ cfu/mL). To inoculate the diluted strain into 1-day 5-instar silkworms, batches of fresh mulberry leaves were prepared to the same weight and were then immersed in a cell suspension at different concentrations (2 × 10^7^ cfu/mL, 3 × 10^8^ cfu/mL, 1.5 × 10^9^ cfu/mL) or PBS buffer. Afterwards, the leaves were fed to silkworms, which were then reared for 7 days, and the mortality rate was recorded. The experiment for each concentration was repeated three times with 50 silkworms each time.

For pathogenicity analysis through injection, 50 μL of 1 × 10^7^ cfu/mL cell suspension was injected into the body cavity using a 36-gauge insulin syringe (BD, Franklin Lakes, NJ, USA); the silkworms were reared for 4 days, and mortality rates were compared.

### 4.6. Statistical Analysis

All experimental results are expressed as mean value ± standard error. Comparisons were made using one-way analysis of variance in IBM SPSS Statistics 23 (IBM Corp., Armonk, NY, USA). The significance of differences was tested by performing Duncan’s multiple rage test. Statistical significance was set at *p* < 0.05.

## 5. Conclusions

Sepsis in silkworms can be caused by various microorganisms, including the newly identified *Serratia* sp., which results in a higher mortality rate than *S. marcescens*, a well-known cause of sepsis, and disrupts the diversity of the silkworm’s microbiome. The results herein suggest that isolated *Serratia* sp. should be considered in disease-cause analysis and prevention planning for industrial insects and potentially developed as a biocontrol agent against insect pests.

## Data Availability

The original contributions presented in the study are included in the article/Supplementary Materials; further inquiries can be directed to the corresponding author.

## Supplementary Materials

The following supporting information can be downloaded at https://www.mdpi.com/article/10.3390/ijms25073957/s1.
