# Supplementary material for: Microbial Community Changes in Silkworms Suspected of Septicemia and Identification of Serratia sp
Source: Int J Mol Sci. 2024 Apr 2;25(7):3957. doi: 10.3390/ijms25073957 (PMC11012086; doi:10.3390/ijms25073957)
Supplement: Supplementary file 1 [file ijms-25-03957-s001.zip › ijms-2932608-supplementary.pdf]

Microbial Community Changes in Silkworms Suspected of Septicemia and Identification of *Serratia* sp.

Supplementary Material

Table S1. Raw data statistics

Sample name	Total read bases (bp)	Total reads	GC (%)	AT (%)	Q20 (%)	Q30 (%)
Norm 1	66,619,728	221,328	52.90	47.10	91.15	81.47
Norm 2	65,049,110	216,110	53.56	46.44	90.76	81.08
Norm 3	45,110,870	149,870	53.05	46.95	90.65	80.97
Sept 1	57,657,754	191,554	55.15	44.85	90.15	80.33
Sept 2	67,107,348	222,948	55.02	44.80	91.05	81.34
Sept 3	58,414,468	222,948	55.21	44.79	91.34	81.77

*Total read bases: Total number of bases sequenced

*Total reads: Total number of reads. For Illumina paired-end sequencing, this value refers to the sum of read 1 and read 2

*GC(%): GC content

*AT(%): AT content

*Q20(%): Ratio of bases that have a phred quality score of over 20

*Q30(%): Ratio of bases that have a phred quality score of over 30

Table S2. Preprocessing summary of trimmed and filtered read counts for each step.

Sample Name	Adapter and primer trimming	Quality filter	Denoised for	Denoised rev	Merged pair	Non-chimeric
Norm 1	107,109	91,969	91,570	91,741	90,786	74,562
Norm 2	104,258	87,783	87,717	87,739	87,510	85,796
Norm 3	71,501	60,519	60,461	60,480	60,335	60,208
Sept 1	92,832	75,956	75,867	75,948	75,848	75,680
Sept 2	108,185	91,166	91,146	91,128	91,064	90,406
Sept 3	94,097	80,268	80,181	80,265	80,125	79,870

Tools for analysis of adapter and primer trimming, Cutadapt; quality filtering, denoising, merging, and chimera removal, DADA2.

Table S3. Sample statistics estimated by ASV analysis corresponding to the six analyzed samples.

Sample Name	ASVs	Chao1	Shannon	Gini-Simpson	Good's Coverage
Norm 1	126.0	126.71	2.16	0.64	0.99
Norm 2	129.0	129.0	2.99	0.73	0.99
Norm 3	81.0	81.0	2.75	0.70	1.0
Sept 1	7.0	7.0	0.66	0.26	1.0
Sept 2	8.0	8.0	0.71	0.2	1.0
Sept 3	6.0	6.0	0.72	0.27	1.0

* ASVs: amplicon sequence variant (ASV) is a term used to refer to single DNA sequences recovered from a high-throughput marker gene analysis.
* Chao1: returns the Chao1 richness estimate for an ASV definition.
* Shannon: the Shannon index takes into account the number and evenness of species.
* Gini–Simpson: the Gini–Simpson index represents the probability that two randomly selected individuals in the habitat will belong to the same species.
* Good's Coverage: coverage is calculated as $C=1-(s/n)$, where s is the number of unique ASVs and n is the number of individuals in the sample. This index provides a relative measure of how effectively the sample represents the larger environment.

```
This_study  GCTGGCGGCAGGCTTAACACATGCAAGTCGAGCGGTAGCACAGGGGAGCTTGCTCCCCGG    60
LC645982.1  GCTGGCGGCAGGCTTAACACATGCAAGTCGAGCGGTAGCACAGGGGAGCTTGCTCCCCGG    60
NR114043.1  GCTGGCGGCAGGCTTAACACATGCAAGTCGAGCGGTAGCACAGGGGAGCTTGCTCCCTGG    60
            ********************************************************* **

This_study  GTGACGAGCGGCGGACGGGTGAGTAATGTCTGGGAAACTGCCTGATGGAGGGGGATAACT    120
LC645982.1  GTGACGAGCGGCGGACGGGTGAGTAATGTCTGGGAAACTGCCTGATGGAGGGGGATAACT    120
NR114043.1  GTGACGAGCGGCGGACGGGTGAGTAATGTCTGGGAAACTGCCTGATGGAGGGGGATAACT    120
            ************************************************************

This_study  ACTGGAAACGGTAGCTAATACCGCATAACGTCGCAAGACCAAAGAGGGGGACCTTCGGGC    180
LC645982.1  ACTGGAAACGGTAGCTAATACCGCATAACGTCGCAAGACCAAAGAGGGGGACCTTCGGGC    180
NR114043.1  ACTGGAAACGGTAGCTAATACCGCATAACGTCGCAAGACCAAAGAGGGGGACCTTCGGGC    180
            ************************************************************

This_study  CTCTTGCCATCAGATGTGCCCAGATGGGATTAGCTAGTAGGTGGGGTAATGGCTCACCTA    240
LC645982.1  CTCTTGCCATCAGATGTGCCCAGATGGGATTAGCTAGTAGGTGGGGTAATGGCTCACCTA    240
NR114043.1  CTCTTGCCATCAGATGTGCCCAGATGGGATTAGCTAGTAGGTGGGGTAATGGCTCACCTA    240
            ************************************************************

This_study  GGCGACGATCCCTAGCTGGTCTGAGAGGATGACCAGCCACACTGGAACTGAGACACGGTC    300
LC645982.1  GGCGACGATCCCTAGCTGGTCTGAGAGGATGACCAGCCACACTGGAACTGAGACACGGTC    300
NR114043.1  GGCGACGATCCCTAGCTGGTCTGAGAGGATGACCAGCCACACTGGAACTGAGACACGGTC    300
            ************************************************************

This_study  CAGACTCCTACGGGAGGCAGCAGTGGGGAATATTGCACAATGGGCGCAAGCCTGATGCAG    360
LC645982.1  CAGACTCCTACGGGAGGCAGCAGTGGGGAATATTGCACAATGGGCGCAAGCCTGATGCAG    360
NR114043.1  CAGACTCCTACGGGAGGCAGCAGTGGGGAATATTGCACAATGGGCGCAAGCCTGATGCAG    360
            ************************************************************

This_study  CCATGCCGCGTGTGTGAAGAAGGCCTTCGGGTTGTAAAGCACTTTCAGCGAGGAGGAAGG    420
LC645982.1  CCATGCCGCGTGTGTGAAGAAGGCCTTCGGGTTGTAAAGCACTTTCAGCGAGGAGGAAGG    420
NR114043.1  CCATGCCGCGTGTGTGAAGAAGGCCTTCGGGTTGTAAAGCACTTTCAGCGAGGAGGAAGG    420
            ************************************************************

This_study  TGGTGAGCTTAATACGCTCATCAATTGACGTTACTCGCAGAAGAAGCACCGGCTAACTCC    480
LC645982.1  TGGTGAGCTTAATACGCTCATCAATTGACGTTACTCGCAGAAGAAGCACCGGCTAACTCC    480
NR114043.1  TGGTGAGCTTAATACGTTCATCAATTGACGTTACTCGCAGAAGAAGCACCGGCTAACTCC    480
            **************** *******************************************

This_study  GTGCCAGCAGCCGCGGTAATACGGAGGGTGCAAGCGTTAATCGGAATTACTGGGCGTAAA    540
LC645982.1  GTGCCAGCAGCCGCGGTAATACGGAGGGTGCAAGCGTTAATCGGAATTACTGGGCGTAAA    540
NR114043.1  GTGCCAGCAGCCGCGGTAATACGGAGGGTGCAAGCGTTAATCGGAATTACTGGGCGTAAA    540
            ************************************************************

This_study  GCGCACGCAGGCGGTTTGTTAAGTCAGATGTGAAATCCCCGGGCTCAACCTGGGAACTGC    600
LC645982.1  GCGCACGCAGGCGGTTTGTTAAGTCAGATGTGAAATCCCCGGGCTCAACCTGGGAACTGC    600
NR114043.1  GCGCACGCAGGCGGTTTGTTAAGTCAGATGTGAAATCCCCGGGCTCAACCTGGGAACTGC    600
            ************************************************************

This_study  ATTTGAAACTGGCAAGCTAGAGTCTCGTAGAGGGGGGTAGAATTCCAGGTGTAGCGGTGA    660
LC645982.1  ATTTGAAACTGGCAAGCTAGAGTCTCGTAGAGGGGGGTAGAATTCCAGGTGTAGCGGTGA    660
NR114043.1  ATTTGAAACTGGCAAGCTAGAGTCTCGTAGAGGGGGGTAGAATTCCAGGTGTAGCGGTGA    660
            ************************************************************

This_study  AATGCGTAGAGATCTGGAGGAATACCGGTGGCGAAGGCGGCCCCCTGGACGAAGACTGAC    720
LC645982.1  AATGCGTAGAGATCTGGAGGAATACCGGTGGCGAAGGCGGCCCCCTGGACGAAGACTGAC    720
NR114043.1  AATGCGTAGAGATCTGGAGGAATACCGGTGGCGAAGGCGGCCCCCTGGACGAAGACTGAC    720
            ************************************************************

This_study  GCTCAGGTGCGAAAGCGTGGGGAGCAAACAGGATTAGATACCCTGGTAGTCCACGCTGTA    780
LC645982.1  GCTCAGGTGCGAAAGCGTGGGGAGCAAACAGGATTAGATACCCTGGTAGTCCACGCTGTA    780
NR114043.1  GCTCAGGTGCGAAAGCGTGGGGAGCAAACAGGATTAGATACCCTGGTAGTCCACGCTGTA    780
            ************************************************************

This_study  AACGATGTCGATTTGGAGGTTGTGCCCTTGAGGCGTGGCTTCCGGAGCTAACGCGTTAAA    840
LC645982.1  AACGATGTCGATTTGGAGGTTGTGCCCTTGAGGCGTGGCTTCCGGAGCTAACGCGTTAAA    840
NR114043.1  AACGATGTCGATTTGGAGGTTGTGCCCTTGAGGCGTGGCTTCCGGAGCTAACGCGTTAAA    840
            ************************************************************

This_study  TCGACCGCCTGGGGAGTACGGCCGCAAGGTTAAAACTCAAATGAATTGACGGGGGCCCGC    900
LC645982.1  TCGACCGCCTGGGGAGTACGGCCGCAAGGTTAAAACTCAAATGAATTGACGGGGGCCCGC    900
NR114043.1  TCGACCGCCTGGGGAGTACGGCCGCAAGGTTAAAACTCAAATGAATTGACGGGGGCCCGC    900
            ************************************************************

This_study  ACAAGCGGTGGAGCATGTGGTTTAATTCGATGCAACGCGAAGAACCTTACCTACTCTTGA    960
LC645982.1  ACAAGCGGTGGAGCATGTGGTTTAATTCGATGCAACGCGAAGAACCTTACCTACTCTTGA    960
NR114043.1  ACAAGCGGTGGAGCATGTGGTTTAATTCGATGCAACGCGAAGAACCTTACCTACTCTTGA    960
            ************************************************************

This_study  CATCCAGAGAACTTTCCAGAGATGGATTGGTGCCTTCGGGAACTCTGAGACAGGTGCTGC    1020
LC645982.1  CATCCAGAGAACTTTCCAGAGATGGATTGGTGCCTTCGGGAACTCTGAGACAGGTGCTGC    1020
NR114043.1  CATCCAGAGAACTTTCCAGAGATGGATTGGTGCCTTCGGGAACTCTGAGACAGGTGCTGC    1020
            ************************************************************

This_study  ATGGCTGTCGTCAGCTCGTGTTGTGAAATGTTGGGTTAAGTCCCGCAACGAGCGCAACCC    1080
LC645982.1  ATGGCTGTCGTCAGCTCGTGTTGTGAAATGTTGGGTTAAGTCCCGCAACGAGCGCAACCC    1080
NR114043.1  ATGGCTGTCGTCAGCTCGTGTTGTGAAATGTTGGGTTAAGTCCCGCAACGAGCGCAACCC    1080
            ************************************************************

This_study  TTATCCTTTGTTGCCAGCGGTTCGGCCGGGAACTCAAAGGAGACTGCCAGTGATAAACTG    1140
LC645982.1  TTATCCTTTGTTGCCAGCGGTTCGGCCGGGAACTCAAAGGAGACTGCCAGTGATAAACTG    1140
NR114043.1  TTATCCTTTGTTGCCAGCGGTTCGGCCGGGAACTCAAAGGAGACTGCCAGTGATAAACTG    1140
            ************************************************************

This_study  GAGGAAGG  1148
LC645982.1  GAGGAAGG  1148
NR114043.1  GAGGAAGG  1148
            ********
```

Figure S1. Sequence alignment analysis. Isolated bacteria (this study), *Serratia nematodiphila* (LC645982.1), *Serratia marcescens* (NR_114043.1) were compared using Clustal Omega for the 16s rRNA region.

A

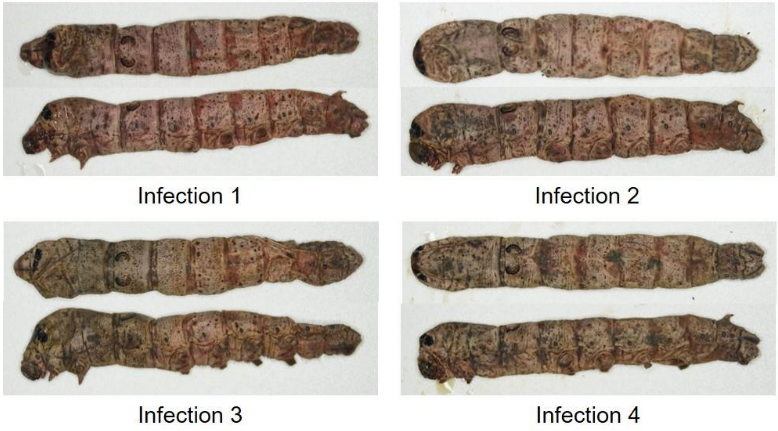

B

```
Serratia sp.      ---------------------------------------------CCAGCAGCCGCGGTA   15
Infection 1       CATCAATTGACGTTACTCGCAGAAGAAGCACCGGCTAACTCCGTGCCAGCAGCCGCGGTA   60
Infection 2       CATCAATTGACGTTACTCGCAGAAGAAGCACCGGCTAACTCCGTGCCAGCAGCCGCGGTA   60
Infection 3       CATCAATTGACGTTACTCGCAGAAGAAGCACCGGCTAACTCCGTGCCAGCAGCCGCGGTA   60
Infection 4       CATCAATTGACGTTACTCGCAGAAGAAGCACCGGCTAACTCCGTGCCAGCAGCCGCGGTA   60
                                                               ***************

Serratia sp.      ATACGGAGGGTGCAAGCGTTAATCGGAATTACTGGGCGTAAAGCGCACGCAGGCGGTTTG   75
Infection 1       ATACGGAGGGTGCAAGCGTTAATCGGAATTACTGGGCGTAAAGCGCACGCAGGCGGTTTG  120
Infection 2       ATACGGAGGGTGCAAGCGTTAATCGGAATTACTGGGCGTAAAGCGCACGCAGGCGGTTTG  120
Infection 3       ATACGGAGGGTGCAAGCGTTAATCGGAATTACTGGGCGTAAAGCGCACGCAGGCGGTTTG  120
Infection 4       ATACGGAGGGTGCAAGCGTTAATCGGAATTACTGGGCGTAAAGCGCACGCAGGCGGTTTG  120
                  ************************************************************

Serratia sp.      TTAAGTCAGATGTGAAATCCCCGGGCTCAACCTGGGAACTGCATTTGAAACTGGCAAGCT  135
Infection 1       TTAAGTCAGATGTGAAATCCCCGGGCTCAACCTGGGAACTGCATTTGAAACTGGCAAGCT  180
Infection 2       TTAAGTCAGATGTGAAATCCCCGGGCTCAACCTGGGAACTGCATTTGAAACTGGCAAGCT  180
Infection 3       TTAAGTCAGATGTGAAATCCCCGGGCTCAACCTGGGAACTGCATTTGAAACTGGCAAGCT  180
Infection 4       TTAAGTCAGATGTGAAATCCCCGGGCTCAACCTGGGAACTGCATTTGAAACTGGCAAGCT  180
                  ************************************************************

Serratia sp.      AGAGTCTCGTAGAGGGGGGTAGAATTCCAGGTGTAGCGGTGAAATGCGTAGAGATCTGGA  195
Infection 1       AGAGTCTCGTAGAGGGGGGTAGAATTCCAGGTGTAGCGGTGAAATGCGTAGAGATCTGGA  240
Infection 2       AGAGTCTCGTAGAGGGGGGTAGAATTCCAGGTGTAGCGGTGAAATGCGTAGAGATCTGGA  240
Infection 3       AGAGTCTCGTAGAGGGGGGTAGAATTCCAGGTGTAGCGGTGAAATGCGTAGAGATCTGGA  240
Infection 4       TGAGTCTCGTAGAGGGGGGTAGAATTCCAGGTGTAGCGGTGAAATGCGTAGAGATCTGGA  240
                  ***********************************************************

Serratia sp.      GGAATACCGGTGGCGAAGGCGGCCCCCTGGACGAAGACTGACGCTCAGGTGCGAAAGCGT  255
Infection 1       GGAATACCGGTGGCGAAGGCGGCCCCCTGGACGAAGACTGACGCTCAGGTGCGAAAGCGT  240
Infection 2       GGAATACCGGTGGCGAAGGCGGCCCCCTGGACGAAGACTGACGCTCAGGTGCGAAAGCGT  240
Infection 3       GGAATACCGGTGGCGAAGGCGGCCCCCTGGACGAAGACTGACGCTCAGGTGCGAAAGCGT  240
Infection 4       GGAATACCGGTGGCGAAGGCGGCCCCCTGGACGAAGACTGACGCTCAGGTGCGAAAGCGT  240
                  ************************************************************

Serratia sp.      GGGGAGCAAACAGGATTAGATACCCTGGTAGTCCACGCTGTAAACGATGTCGATTTGGAG  215
Infection 1       GGGGAGCAAACA------------------------------------------------  312
Infection 2       GGGGAGCAAACA------------------------------------------------  312
Infection 3       GGGGAGCAAACA------------------------------------------------  312
Infection 4       GGGGAGCAAACA------------------------------------------------  312
                  ************
```

Figure S2. Microbial identification from silkworms re-infected with the isolated bacteria. (A) Among the silkworms re-infected with the isolated bacteria, four silkworms showing signs of disease were randomly selected. (B) Bacteria from the selected silkworms were extracted using PBS and plated on LB medium, and the most abundant morphological colonies were selected and the 16S rRNA gene sequence (the size of about 312 bp) was amplified using primers 518F and 907R and sequenced for comparison with the sequence of the infecting bacteria.
